# Control of Relative Timing and Stoichiometry by a Master Regulator

**DOI:** 10.1371/journal.pone.0127339

**Published:** 2015-05-22

**Authors:** Yifat Goldschmidt, Evgeny Yurkovsky, Amit Reif, Roni Rosner, Amit Akiva, Iftach Nachman

**Affiliations:** Department of Biochemistry and Molecular Biology, Tel Aviv University, Tel Aviv, Israel; University College London, UNITED KINGDOM

## Abstract

Developmental processes in cells require a series of complex steps. Often only a single master regulator activates genes in these different steps. This poses several challenges: some targets need to be ordered temporally, while co-functional targets may need to be synchronized in both time and expression level. Here we study in single cells the dynamic activation patterns of early meiosis genes in budding yeast, targets of the meiosis master regulator Ime1. We quantify the individual roles of the promoter and protein levels in expression pattern control, as well as the roles of individual promoter elements. We find a consistent expression pattern difference between a non-cofunctional pair of genes, and a highly synchronized activation of a co-functional pair. We show that dynamic control leading to these patterns is distributed between promoter, gene and external regions. Through specific reciprocal changes to the promoters of pairs of genes, we show that different genes can use different promoter elements to reach near identical activation patterns.

## Introduction

Many processes which involve a major change in the state of the cell (such as differentiation, meiosis and sporulation) are governed by a master regulator in the form of a single transcription factor that activates dozens or even hundreds of the genes needed to carry out the process [1–4]. Such regulation architecture poses two challenges. First, different genes may need to be correctly timed, i.e. activated at the right stage of the process [5]. Second, groups of co-functional genes, i.e. genes that collaborate on the same function, such as complex members, may need to be tightly synchronized, as any deviation from the correct relative stoichiometry may result in unused proteins and possible toxic effects from partial complexes. It is not known to what extent these two expression properties, relative timing and correct stoichiometry, are regulated in each cell. It is also not clear whether the master regulator alone is responsible for timing and synchrony (through transcription regulation), or whether post-transcriptional stages, such as differential protein and mRNA stability or protein localization, play a major role.

There is yet limited knowledge on what mechanisms and genomic elements contribute to differential timing and stoichiometry of jointly regulated genes. Previous works have shown the existence of regulated target timing in temporal processes. Differential timing has been shown in bacteria in multiple regulator cascades [5,6] and within the SOS regulon controlled by a single regulator [7]. In eukaryotic systems, several studies used artificial variants of one promoter [8–10]. Lam *et*. *al*. [8] showed that in the PHO5 promoter, containing two binding sites for Pho4, the exposed binding site controls activation timing while the nucleosome-occluded site controls maximal expression level. Sharon *et*. *al*. [9], studied thousands of promoter variants, finding a relationship between expression and binding site multiplicity, as well as a TF-specific dependency of expression on binding site distance from gene start. Eser *et*. *al*. [11] found that the activation time of G1/S target genes depends on combinatorial regulation by the MBF and SBF cell cycle regulators. Zeevi *et*. *al*. [12] found that proper stoichiometry of ribosomal protein (RP) expression is at least partly encoded in the promoters through the amount of nucleosome-disfavoring sequences, as well as characteristic spatial organization of those sequences and of binding sites for key RP regulators.

**Fig 1:**
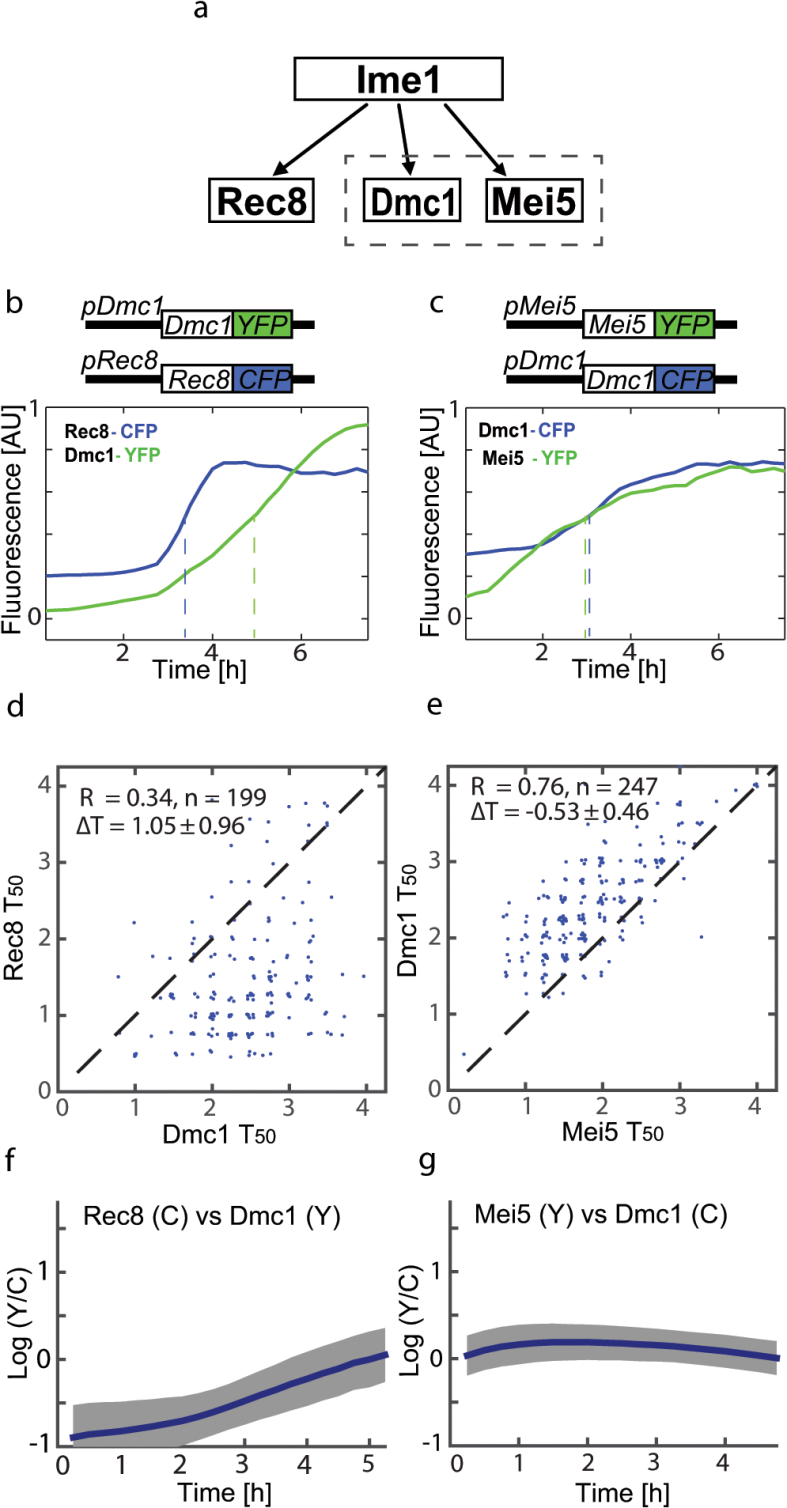
Co-regulated protein pairs show different levels of coordination. (a) The regulation scheme of Rec8, Dmc1 and Mei5 by Ime1. The dashed box illustrates the joint function of Dmc1 and Mei5. (b,c) Single cell examples for protein levels over time: Rec8-CFP, Dmc1-YFP (b), Dmc1-CFP, Mei5-YFP (c). Dashed lines mark T_50_ for each marker (see text). Difference in the initial expression level between CFP and YFP comes from the higher signal to noise ratio of the CFP signal, see S1D and S1E Fig. T_50_ values for protein fusion cells with Dmc1, Rec8 (d) or Dmc1, Mei5 (e) markers. In all plots, y-axis marker is CFP, x-axis is YFP. For each plot, the mean and std of ΔT_50_ (the distance from the diagonal) are shown. (f,g) Relative abundance over time, measured as log(YFP/CFP) in single cells for the two pairs. Solid line and gray area show mean and standard deviation. Cells were aligned by onset time (see text). For better estimation of variability, the genes are tagged on both chromosomal copies. The co-functional pair (Dmc1, Mei5) displays higher coordination (both in relative abundance and in timing) than the non co-functional pair (Dmc1, Rec8).

Here, we use a single cell comparative approach to study the questions of relative target timing and stoichiometry. We use early meiosis in yeast as a model for a system with a master regulator. Ime1, the master regulator of early meiosis, is controlled by multiple starvation signals [13–15]. Through its interaction with Ume6, Ime1 activates many genes involved in the early steps of meiosis [16–19]. These early meiosis genes include Rec8, Dmc1 and Mei5 (Fig 1A). Rec8 is a meiosis specific part of the cohesin complex, responsible for sister chromatid cohesion, but also playing independent roles in homolog pairing, recombination, chromosome axis formation and synaptonemal complex assembly [20–22]. Dmc1 is a RecA homolog, playing a role in homology search and strand exchange during meiotic recombination [23]. Its activity depends on the heterodimeric mediator complex Mei5-Sae3 [24]. Dmc1 and Mei5 co-localize in a mutually dependent manner on meiotic chromosomes, and play a co-dependent role in the same meiotic phase [25]. We chose to focus on the expression patterns of Rec8, Dmc1 and Mei5, as they form a co-functional pair (Mei5 and Dmc1) and a non co-functional pair (Rec8 and Dmc1). Their known functions imply that Rec8 is required in the cell before Dmc1 and Mei5, which are in turn required at the same time. All three of these targets have no known regulatory effects on Ime1, on themselves, or on each other, and therefore may represent open-loop (no feedback) dynamics generated by a transcription factor.

We find that the co-functional pair displays a tight synchrony with a small offset at the single cell level, while the non co-functional pair is differentially timed and un-synchronized. We show that timing and stoichiometry control are regulated through different parts of the gene, including ORF, promoter and regions outside the promoter, and that different genes can attain very similar expression profiles through different regulatory elements.

## Materials and Methods

### Media, yeast strains

All strains are from the SK1 background [26]. Transformations were done using the lithium-acetate method. Protein fusions were done by inserting a yeast-codon optimized ORF of a fluorescent protein (Venus, Citrine, Cerulean or CFP, [27]) in frame before the gene stop codon, followed by a selection marker. Promoter fusions were done by replacing the WT ORF (from ATG to stop codon) with the ORF of a fluorescent protein, preceded by a nuclear localization signal (NLS) and followed by a selection marker.

Gene swap and promoter-gene swap strains were created as follows: for gene swap, the ORF-XFP-resistance-marker segment of Rec8 (Dmc1) was PCR-amplified from a protein-fusion strain using primers with 40 bp tails homologous to the regions upstream of Dmc1 (Rec8) gene start and downstream of its stop codon. This linear segment was then transformed to a wild type strain, replacing the wild type Dmc1 ORF. For promoter-gene swap, a similar procedure was carried, but amplifying the region starting from the promoter (downstream of the next ORF, 473 bp upstream of ATG for Rec8, 341 bp upstream of ATG for Dmc1). In that case the 40bp homology in the 5’ primer matched the promoter start of the opposite gene.

Specific motif changes were made by cloning of the target ORF from the tagged yeast strain, starting at a position immediately after the region to be changed, and then extending this construct by a forward primer containing the required change at a length of ~40 bp and a complementary portion of ~20 bp, and a reverse primer from the previous reaction.

All Dmc1 promoters with motif changes were tagged with Venus, and mated with a native Dmc1 strain tagged with Cerulean. All Rec8 promoters with motif changes were tagged with Cerulean, and mated with a native Rec8 strain tagged with Venus. Mei5 motif change was done similarly.

### Microfluidic Experiments

Microfluidic chambers were made from Poly-dimethylsiloxane (PDMS) polymer using SYLGARD 184 silicone elastomer kit (Dow Corning, Midland MI, USA) and a 1:10 curing agent ratio. Monomer base and curing agent mix was poured onto a photoresist mold and then incubated overnight at 65°c, forming simple channels each with one input and output. The PDMS was then reversibly attached to a cover glass slide by pre-treatment with a hand-held plasma generator. In order for cells to adhere to the bottom of the glass, the channels were then filled with a 2mg/ml Concanavalin A solution supplemented with 5mM CaCl_2_ and 5mM MnCl_2_ in sterile double distilled water.

Cells were picked from single colonies and grown overnight before each experiment in YPD (1% Bacto yeast extract, 2% Bacto peptone, 2% glucose). The cells were then washed twice with SPM medium (0.4% Potassium Acetate, 0.02% raffinose) at 2500 rpm for 1 minute. Washed cells were then inserted into the channels to form a single cell layer. SPM medium was pumped into each channel at a constant rate of 0.5 ml/hour. Images were taken using a Nikon TiE fluorescent microscope at 100X or 60X magnification. In each experiment 10 positions along the channel containing a total of 300–1000 cells were chosen. These positions were imaged every 15 minutes using the relevant fluorescent emission and detection filters (490/535 for YFP and Venus, 400ms–600ms exposure; 435/465 for CFP and Cerulean, 1–2 sec exposure).

### Image analysis

The captured images were analyzed using custom written code [28] in Matlab (Mathworks, Inc.). Nuclear signal level for YFP and CFP was defined as the mean over the 15 brightest pixels within the cell. Cell background was defined as the median of the 30% dimmest pixels within the cell. After subtracting cell background and per-experiment residual background from nuclear signal, photobleaching correction was performed using experimentally derived photobleaching coefficients. CFP and YFP signals were then scaled by their per-experiment maximum, except when comparing promoter variants where scaling factors were chosen to set the wt-CFP vs. wt-YFP at similar levels, and were then fixed for all variants of that promoter.

### Promoter sequence analysis

Genetic sequence data was obtained from the UCSC genome browser (http://genome.ucsc.edu/) and from GermOnline (http://www.germonline.org/index.html). Expression data during meiosis was obtained from Brar *et al* [29]. A list of Ume6 targets was obtained from Williams *et al* [19]. The Ume6 binding site PSSM model from MacIsaac *et al* [30] was scanned against the target promoters binding site locations. The genes containing this site were selected for further analysis. Nuc +1 and Nuc -1 location data was obtained from Tsankov *et*. *al*. [31] describing both ends of the NFR for all yeast genes. 5’UTR data was obtained from Lin *et*. *al*. [32]. TATA box data was obtained from Basehoar *et*. *al*. [33]. TSS data was obtained from Miura *et. al. [34]*.

## Results

### Co-regulated targets can be differentially timed or highly synchronized

To study synchrony and differential timing in targets of the same master regulator, we measured the single-cell expression dynamics of Rec8, Dmc1 and Mei5 during the induction of meiosis in diploid yeast cells (Fig 1A). In each experiment two proteins were tagged within the same cells using fluorescent proteins, and their nuclear accumulation was monitored using time-lapse microscopy in microfluidic devices (S1 Movie). The double tagging allows for measurements of two expression profiles that are affected by the exact same cellular conditions, such as transcription factor concentration, polymerase concentration or ribosome abundance (S1 Fig). We used here the common C-terminal tagging [11,35,36] that eliminates the endogenous 3’ UTR regions of the genes, which could potentially affect their mRNA stability.

We first quantify the timing difference and synchrony between two targets of a master regulator having distinct functions. To this end, we examined Rec8 and Dmc1, as these proteins work at different phases of early meiosis and are both targets of Ime1. Rec8 activity is needed before that of Dmc1, as it participates in earlier phases of the process (e.g. sister chromatid cohesion). We followed single cells after induction of meiosis in a diploid strain in which the Dmc1 protein is fused to YFP and Rec8 protein is fused to CFP (Fig 1B). Rec8 and Dmc1 display different activation patterns at single cell resolution (Fig 1B). Rec8 levels consistently rise to their maximal level faster than Dmc1 at the early stages, and often stop accumulation while Dmc1 continues to rise in a roughly linear manner. To examine two proteins that work together on the same function we tagged Dmc1 and Mei5 in the same cells. The results showed that the temporal patterns of these proteins are very similar (Fig 1C). To compare protein activation times, we define an “onset time” for each cell as the time at which the earlier of the two proteins reaches 20% of its maximal level. This serves as an approximation for the onset of the transcription program induced by Ime1, which is highly variable between cells [28]. We then focus on the following 7 hours, and denote by T_50_ the time at which each of the proteins reaches 50% of its maximal level over the 7 hour period. The definition, based on a cell-internal clock, allows the more accurate measurement of relative activation between pairs of targets. This measure is robust against different working levels for different proteins, and for the same protein over different cells due to variable cell specific parameters. Comparing the T_50_ of the two protein pairs reflects the observation from the single cell tracks, showing variable time delay between Dmc1 and Rec8 (mean 1.0h, sd 0.9h, Fig 1D) and a smaller and tighter time difference between Mei5 and Dmc1 (mean -0.5h, sd 0.45h, Fig 1E).

We next quantified the coordination in protein levels of the gene pairs. The protein products of a pair of genes that are involved in the same function may play their role at a certain stoichiometry (for example, if they are complex members). In such a case, it may be beneficial to regulate not only the relative timing of those genes but also their relative stoichiometry. We therefore wanted to check whether the co-functional pair of proteins (Dmc1 and Mei5) has a tighter stoichiometry at the protein level than the non co-functional pair (Dmc1 and Rec8). We note that tight stoichiometry does not automatically follow from coordinated timing: theoretically, a pair of proteins can be highly synchronized in their timing (as are Dmc1 and Mei5), yet have high cell-to-cell variability in their relative abundance. Examining the log ratio of the two signals over time, we find that the protein level ratio between Mei5 and Dmc1 stabilizes on a constant value within a few hours from onset, with a tighter distribution over time, while the ratio between Dmc1 and Rec8 changes over time (Fig 1F and 1G). This divergence, likely a direct result of differences in temporal profiles and activation times in these two genes, suggests their respective regulatory mechanisms were not optimized to maintain a certain protein ratio.

Overall, the two pairs defined by these 3 genes show distinct relations in their protein levels, where one pair is differentially timed and divergent in relative abundance, and the other more synchronized and tightly coordinated in stoichiometry.

### Effects of individual promoter elements on timing and stoichiometry

**Fig 2:**
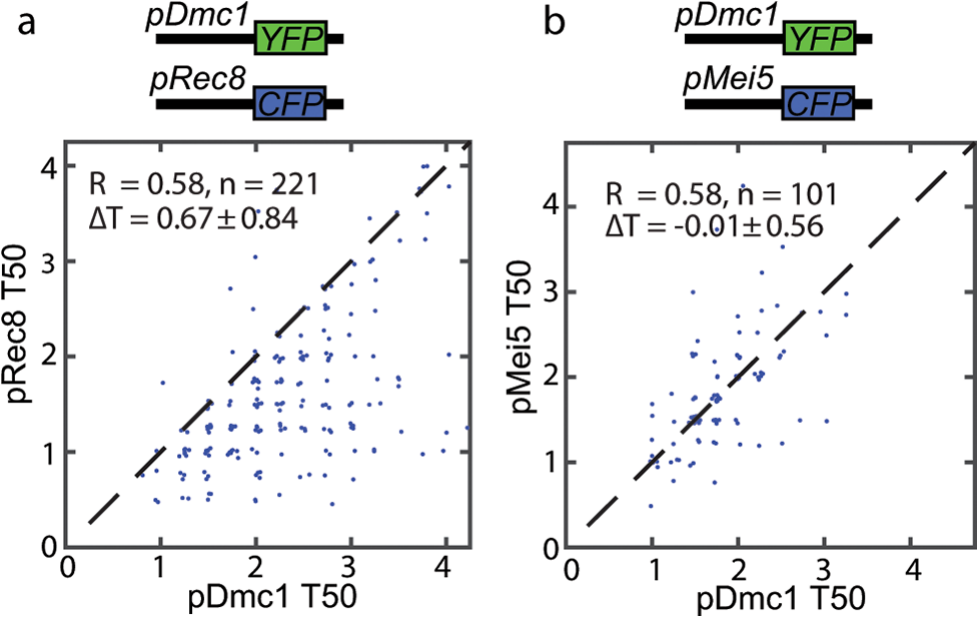
Protein pair coordination level is reflected at the promoter level. Shown are T_50_ values for promoter fusion cells with pDmc1, pRec8 (a) or pDmc1, pMei5 (b) markers. The pair (Dmc1, Mei5) shows higher coordination than (Dmc1, Rec8) at the promoter activity level.

We next looked for the regulation level at which the activation and de-activation timing are controlled. Since we measure nuclear protein levels, this regulation could occur at the level of transcription, translation, protein localization, mRNA or protein degradation or any combination of the above. To study the role of promoter activity in the protein expression pattern, we tagged the promoters of Rec8, Dmc1 and Mei5 with fluorescent proteins (replacing the endogenous ORF with NLS-CFP or NLS-YFP). The results again showed a consistent difference between Rec8 and Dmc1, with a smaller temporal offset compared to the protein level (Fig 2A). The Dmc1 and Mei5 promoters presented a similar activation profile in each cell, as did their protein level profiles (Fig 2B), but the temporal offset seen at the protein level is gone.

Our results suggest that there is a major contribution to the regulation of timing of our test gene pairs at the transcriptional level. If specific promoter features regulate timing and expression level similarly in many targets of the Ime1 master regulator, we expect to identify a correlation between such features and the expression properties [12]. We therefore analyzed the promoter regions of 71 genes with known Ume6 binding sites alongside their mRNA expression profiles [29]. Distances between different promoter elements (TATA box, Ume6 binding site, ORF start, +1 and -1 nucleosome) were correlated to either expression level or timing (S2 Fig). While most distances did not correlate with either expression level or timing, we found a weak connection between the location of the TATA box and the maximal mRNA level (S2C Fig). Generally, in "strong" promoters the TATA box (if present) is closer to the ATG, while in "weak" promoters it is farther upstream from the ATG and spread over a larger range. These results however, did not help us in forming hypotheses on our genes of interest (Rec8, Dmc1, Mei5), as the difference between these genes in each of the control elements are on a smaller scale than the effect found by the large scale analysis. Moreover, no single promoter attribute was found to correlate with activation timing across the set of Ume6 targets genes.

**Fig 3:**
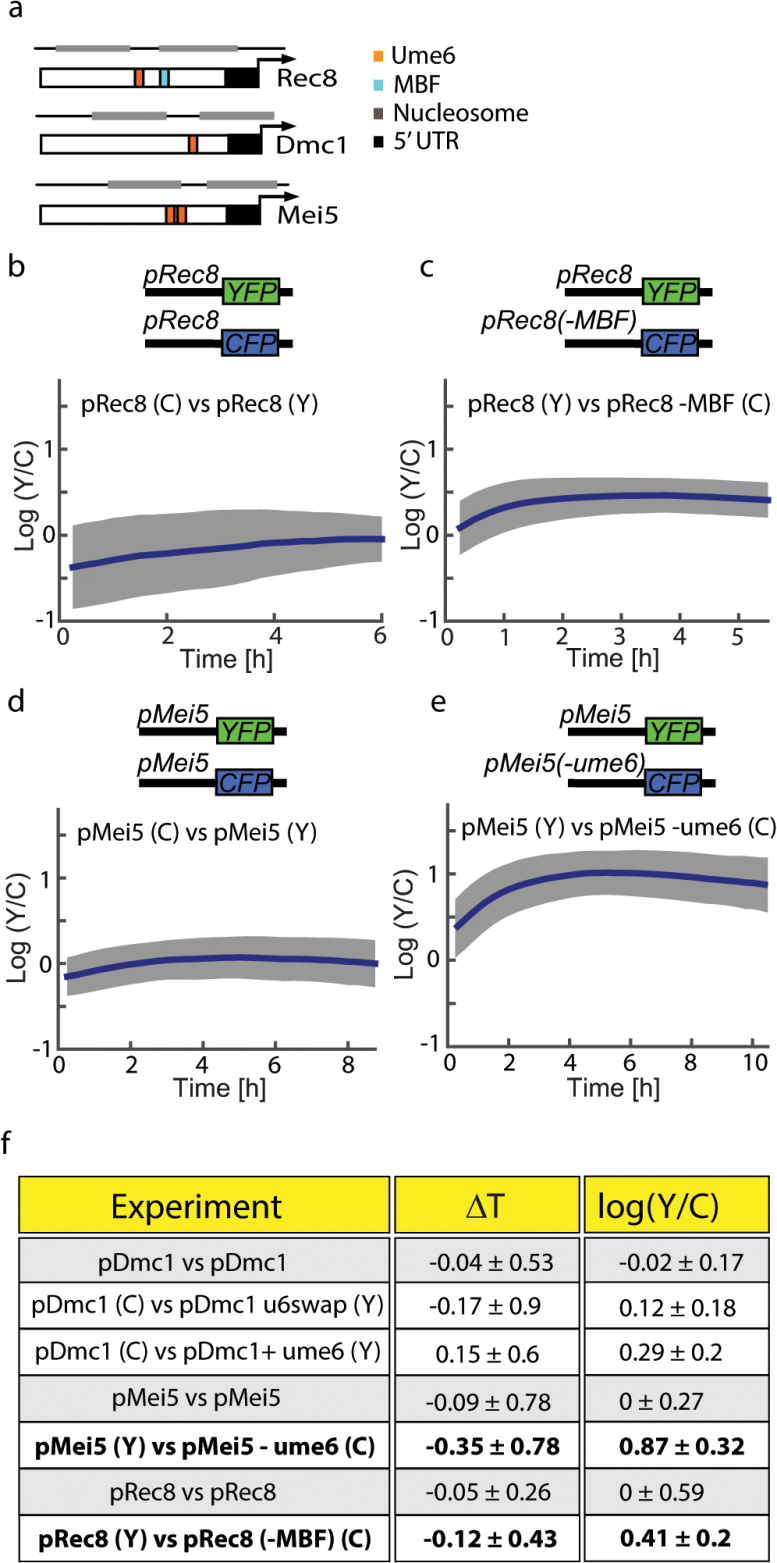
Effects of single binding site changes on absolute levels, timing or variability of stoichiometry. (a) A scheme of the promoters of Rec8, Dmc1 and Mei5. Gray bars denote the predicted +1 and -1 nucleosome locations. (b,c) Relative abundance over time in single cells of the pRec8-YFP, pRec8-CFP (b) and pRec8-YFP, pRec8(-MBF_bs_)-CFP (c) promoter fusion strains. (d, e) Relative stoichiometry over time in single cells of the pMei5-YFP, pMei5-CFP (d) or pMei5-YFP, pMei5(-UME6_bs_)-CFP (e) strains. (f) A summary of the motif change experiments (see also S3 Fig). For each comparative experiment the mean and standard deviation of ΔT_50_ and of *log(YFP/CFP)* in the terminal time point are shown. Removal of MBF binding site decreases pRec8 activity uniformly, with no significant effect on activation timing. Removal (or addition) of a second Ume6 binding site from pMei5 (or pDmc1) also affects mostly the absolute level.

We then attempted to narrow down the specific promoter elements that dominate the temporal behavior of Rec8, Dmc1 and Mei5 (Fig 3A). We decided to focus on the effects of specific changes to binding sites in previous studies showed that sequence changes in transcription factor binding sites in the promoter give rise to different gene expression profiles [37]. We therefore wanted to test if Rec8 contains an MBF binding site in a nucleosome-covered region. Since the components of MBF (Mbp1 and Swi6) are activated in early meiosis [17,38] one hypothesis would be that MBF binds its meiotic targets once the occluding nucleosome is cleared (possibly by Ime1 or remodelers recruited by it [39]), and then modulates their activation. Another possibility would be the difference in Rec8 and Dmc1's Ume6 binding site, which differ in 2 out of 10 positions.

To test the effect of individual sequence changes in the various promoters, we created strains with one WT allele and one altered allele of that gene. This allowed the accurate comparison of the *cis*-effect of the altered allele to the wild type in individual cells, where the *trans* environment (including Ime1 protein concentration) is identical. This was important, as the cell-to-cell variability between Ime1 profiles and target promoter activation was high [28].

The perturbation of the MBF binding site located on the Rec8 promoter resulted in a marked reduction of Rec8 promoter activity, compared to the WT Rec8 promoter (Fig 3B and 3C). This reduction is detectable throughout the entire duration of the experiment, suggesting that MBF has a positive effect on Rec8 expression throughout its activation period in meiosis. Notably, this mutation has no significant effect on the activation time (or T50) for pRec8 (S2C Fig, p = 0.34). Changing the Ume6 binding site composition on the Dmc1 promoter to the composition of its site on the Rec8 promoter showed no noticeable change in Dmc1's expression pattern compared to the WT Dmc1 allele (S3A Fig). We next probed what elements may affect the similarity in expression dynamics of Mei5 and Dmc1. This similarity is interesting as these two genes have distinct differences in their promoter composition. One major difference is the presence of two Ume6 binding sites (each with a different composition) on the Mei5 promoter, while Dmc1's promoter only contains one such binding site. Previous works have shown that two different binding sites in the same promoter (that may differ in their affinity or in occlusion by nucleosomes) can have distinct contribution to the onset time and to the maximal promoter activity [8,9]. In order to analyze this difference we first perturbed one of the binding sites on pMei5 and measured the effect compared to the WT Mei5 allele. This resulted in a significant reduction in Mei5 promoter activity (Fig 3D and 3E), with only a small effect on activation timing (S3B Fig). Alternatively, adding a Ume6 binding site to the Dmc1 promoter 7 base pairs upstream of the existing one (to maximize the similarity to the Mei5 promoter) resulted in a small increase in promoter activity and slightly earlier activation time (S3C Fig). In summary, most single motif changes we attempted had no or little effect on activation timing, while the addition or removal of a TF binding site had a significant effect on promoter activity (Fig 3F).

### ORF and promoter contribute independently and consistently to timing and level of Rec8 and Dmc1

The lack of significant contribution of individual promoter element changes to the timing regulation stands in contrast to our observation that the timing is highly reflected in the promoter activity level. This suggests that the timing of these genes is controlled by other promoter elements, by a combined effect of several promoter elements, by elements outside the promoter or by a combination of the above. Moreover, the timing difference between the promoters (Fig 2) is smaller than the timing difference at the nuclear protein level (Fig 1D and 1E), suggesting the ORF identity has a significant effect on timing, through one or more of the possible post-transcriptional mechanisms. In order to determine the relative contribution of the ORF, the promoter and regions external to the promoter and ORF we have created alleles in which these three types of segment appear in different combinations. To this end we replace the ORF (or the ORF and promoter) of Rec8 with the ORF (or the ORF and promoter) of Dmc1, and vice versa. This resulted in a series of 6 alleles, denoted by DDD, DDR, DRR, RRR, RRD and RDD, where the three letters denote (from left to right) the external, promoter and ORF regions. For example, the strain RDD contains the promoter and ORF regions of Dmc1 replacing the promoter and ORF regions of Rec8. We have created diploid strains with pairs of these 6 alleles, to make direct comparisons between alleles differing in one or more of the regions, thus isolating the effects of specific regions. For example, the strain RRD-YFP, RRR-CFP isolates the contribution of ORF on timing (in the context of Rec8 external and promoter regions).

**Fig 4:**
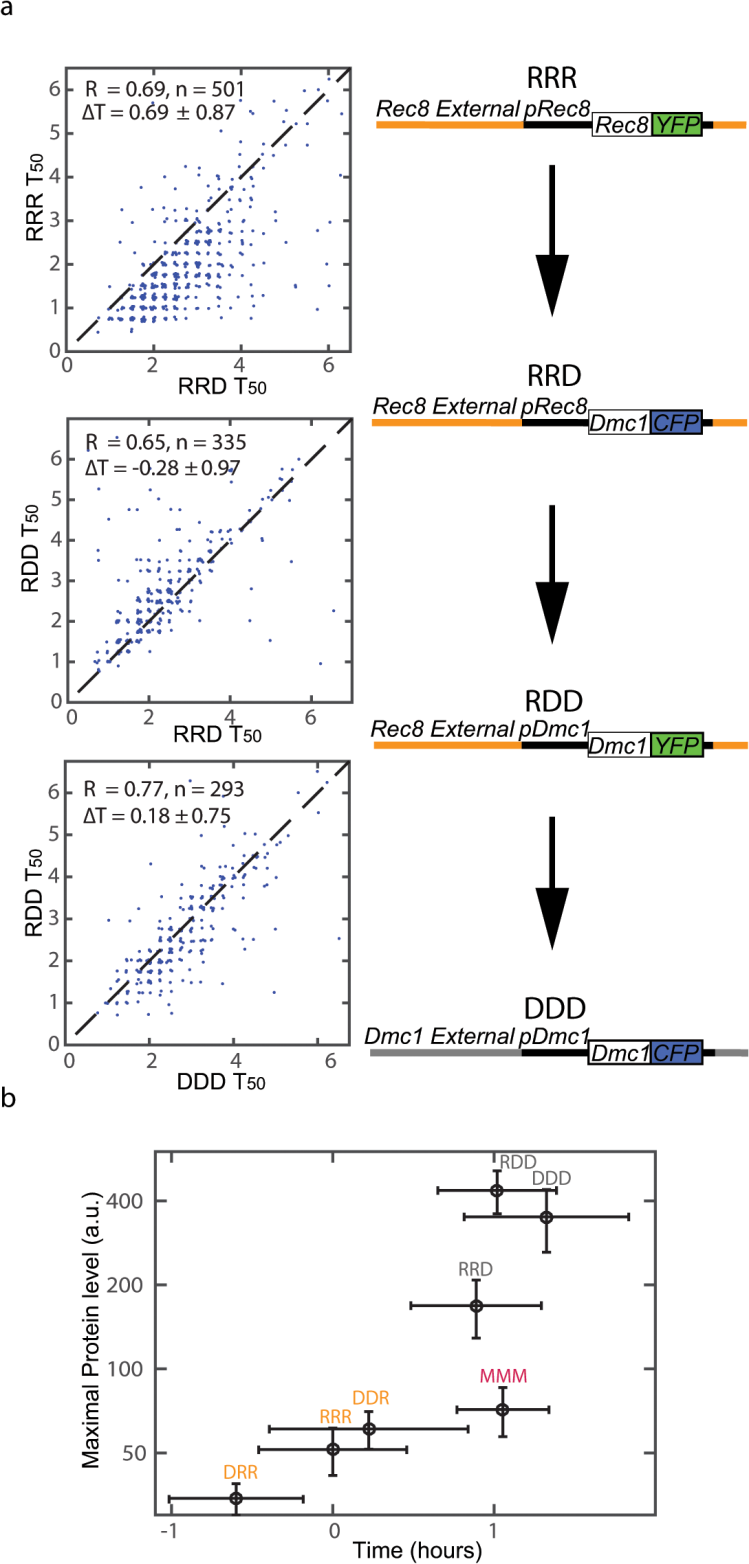
Contributions of ORF, promoter and external regions to timing and maximal protein levels. (a) T_50_ values along a path from RRR to DDD alleles, where each step compares two alleles that differ in one region (ORF, promoter or external). The dominant contribution to timing difference comes from the ORF level (RRR→RRD). (b) A summary of comparative experiments between pairs of the 6 alleles and the Mei5 (MMM) allele. For each allele, the maximal protein level is plotted against its T_50_ relative to the other alleles (where RRR was placed at t = 0). Both maximal level and relative timing were averaged over multiple pairwise experiments.

Fig 4A shows the relative activation times of pairs of alleles along a path from RRR to DDD, where each step compares two alleles that differ in one region (ORF, promoter or external). The dominant contribution to timing difference comes from the ORF level (RRR→RRD), where the ORF of Dmc1 delays activation. The change of promoter (RRD→RDD) and external regions (RDD→DDD) delay the activation time as well, albeit to a smaller extent.

We now compare the 6 alleles in terms of their relative timing and maximal protein expression level during the 7 hours period starting from the onset time. To this end, we integrate the relative timing and abundance information from single experiments into one comparative view. Fig 4B shows this comparison, alongside the Mei5 (or MMM) allele. Comparing the three wild type alleles (MMM, RRR and DDD) shows their abundance ratios in addition to their temporal relations we already observed in Fig 1. While Mei5 (MMM) activation time closely precedes that of Dmc1, its expression level is about 5-fold lower, consistent with its suggested functional roles as a mediator of Dmc1 filament assembly during meiotic recombination [25]. We can use the three wild type alleles as the anchor points to which we compare the behaviors of the other permutation alleles.

First, we note that behavior of the alleles clusters according to the ORF identity, as the ORF provides the dominant contribution to both timing and maximal protein level. Second, the promoter of Dmc1 leads to delayed activation and increased maximal level in two different contexts (RRD→RDD and DRR→DDR). Therefore its contribution to timing and level is consistent with that of the Dmc1 ORF. Finally, the external regions at the Rec8 locus contribute to higher expression (RDD→DDD and RRR→DRR), but their effect on activation time depends on the promoter or gene context: the external regions of the Dmc1 locus contribute to earlier activation of a Rec8 gene (RRR→DRR), but not of Dmc1 (RDD→DDD).

## Discussion

We have studied the degree of differential or synchronous timing and stoichiometry in single cells in two examples of gene pairs controlled by the same transcription factor. We used live imaging of single diploid *S*. *cerevisiae* cells containing either protein or promoter tagging of pairs of Ime1 target genes. Our results show that these target genes can be more tightly synchronized and settle on a set relative stoichiometry (Dmc1 and Mei5) or have a relative delay with variable lag between one another, leading to a change over time in relative abundance (Dmc1 and Rec8). The Dmc1 and Mei5 pair showed tight synchronization and no temporal offset at the promoter level, only gaining an offset at the protein level. In contrast, the Rec8 and Dmc1 pair showed temporal offset and high variability both at the protein and the promoter levels.

Our bioinformatic analysis of Ume6 targets does not show a correlation of any of the promoter parameters tested with expression timing, and only a few distance parameters showed some correlation to maximal expression level. This suggests that the shape of gene activation patterns may result from a combination of several control elements, or the utilization of different elements in each promoter. Consistently, Dmc1 and Mei5, which have a very tightly synchronized protein level pattern, have two very different promoter sequences and gene properties (the ORF of Dmc1 is three times longer and contains an intron). Removing one of the Ume6 binding sites from pMei5 or adding such a binding site to pDmc1 caused a decrease or increase (respectively) in promoter activity, but had little effect on activation timing. This suggests that Dmc1 and Mei5 have co-evolved to increase their synchrony, each using a different combination of specific promoter and gene elements. The similar reduction in Rec8 promoter activity upon removal of the nucleosome-covered MBF binding site indicates that MBF acts as a co-activator for this gene, and probably only binds to its binding site once the nucleosome occluding it has been removed, increasing Rec8's transcription rate. The effect on timing, however, is small. This outcome is similar to the effect of the second Ume6 binding site for Mei5: in both cases, the additional binding site serves mostly to increase maximal expression levels, with little effect on timing, consistent with previous results [8]. The utilization of a different transcription factor (MBF) in this role is a specific, rather than a general mechanism in this gene module (only one other Ume6 target gene (YOR1) has an MBF binding site in its promoter).

Dissecting the contributions of ORF, promoter and external regions to the timing and maximal level of Rec8 and Dmc1 lead to several insights. First, it is the ORF that contributes mostly to the timing and maximal working level in these two genes. This may be mediated through the stability of the mRNA, protein or both, as increased stability in either level can lead to slower accumulation as well as higher maximal expression level. The contribution of the promoter region to these properties is secondary, but it is consistent with the ORF contribution: both promoter and ORF contribute independently to later activation and higher maximal level in Dmc1. Second, the contribution of the external regions, providing the chromosomal context in which the gene is located, depends on the gene (or promoter) and varies between the two genes.

Together, our results show that yeast can attain both remarkable synchronization and differential timing between protein pairs controlled by the same regulator, using very different mechanisms and cis-elements for each gene. The genes tested in this work are activated by a regulator that interacts with the DNA through a co-factor (Ume6). It would be interesting to examine target genes of a different type of regulator, one that binds directly to the DNA, and see whether it produces different dynamical effects on its targets.

## Supporting Information

S1 Fig CFP and YFP signals from an example single cell. Normalized CFP and YFP time series from a single Mei5-CFP, Dmc1-YFP cell entering meiosis. Four snapshots are shown from the first 3 hours after switch to meiotic conditions. YFP has a better signal to background ratio than CFP, most apparent in the early part of the series, where the signals are weak. As a result, the early ramp-up part in the CFP channel is below detection level. The T_50_ statistic is designed to overcome the effect of this artifact.
(EPS)

S2 Fig Effect of promoter sequence elements on expression timing and maximal level. (a,b) Promoter architecture and normalized mRNA expression profiles of Ime1 target genes. The genes are aligned by ATG and sorted by expression peak time (a) or by maximal expression level (b). Ume6 binding site—red; TATA box—blue; TrSS—green; measured nucleosomes—gray. (c, d) Distributions of TATA box (c) or Ume6 binding site (d) distance to ATG for different expression level groups (as marked in (b)). Triangles show th 95% confidence interval for the median estimator. In highly expressed genes the TATA box is closer to the ATG.
(EPS)

S3 Fig Individual binding site changes mostly affect expression level. Timing and stoichiometry measures for strains having one alteration in the promoter of Dmc1, Rec8 or Mei5, compared to the wild type promoter. For each strain, scatter plots of T_50_ and tracks of log(YFP/CFP) over time (mean, std) are shown. (a) pDmc1 vs. pDmc1 (left), pDmc1 vs. pDmc1(UME6bsSwap) (center), pDmc1 vs. pDmc1+UME6bs (right). (b) pMei5 vs. pMei5 (left), pMei5 vs. pMei5-UME6bs (right). (c) pRec8 vs. pRec8 (left), pRec8 vs. pRec8-MBFbs (right).
(EPS)

S1 Movie Time lapse imaging of dual color cells entering meiosis. Part of a single position time-lapse movie showing Rec8-CFP, Dmc1-YFP cells entering meiosis. Left: Rec8-CFP signal. Right: Dmc1-YFP signal. Center: merged CFP, YFP and DIC channels. Time interval between acquisitions: 15 minutes.
(AVI)

## References

[1] Fujita M, Gonzalez-Pastor JE, Losick R (2005) High- and low-threshold genes in the Spo0A regulon of Bacillus subtilis. J Bacteriol 187: 1357–1368. 15687200 10.1128/JB.187.4.1357-1368.2005 PMC545642

[2] Mandel S, Robzyk K, Kassir Y (1994) IME1 gene encodes a transcription factor which is required to induce meiosis in Saccharomyces cerevisiae. Dev Genet 15: 139–147. 8205723 10.1002/dvg.1020150204

[3] van Sinderen D, Luttinger A, Kong L, Dubnau D, Venema G, Hamoen L (1995) comK encodes the competence transcription factor, the key regulatory protein for competence development in Bacillus subtilis. Mol Microbiol 15: 455–462. 7783616 10.1111/j.1365-2958.1995.tb02259.x

[4] Little JW, Mount DW (1982) The SOS regulatory system of Escherichia coli. Cell 29: 11–22. 7049397 10.1016/0092-8674(82)90085-x

[5] Zaslaver A, Mayo AE, Rosenberg R, Bashkin P, Sberro H, Tsalyuk M, et al (2004) Just-in-time transcription program in metabolic pathways. Nat Genet 36: 486–491. 15107854 10.1038/ng1348

[6] Kalir S, McClure J, Pabbaraju K, Southward C, Ronen M, Leibler S, et al (2001) Ordering genes in a flagella pathway by analysis of expression kinetics from living bacteria. Science 292: 2080–2083. 11408658 10.1126/science.1058758

[7] Ronen M, Rosenberg R, Shraiman BI, Alon U (2002) Assigning numbers to the arrows: parameterizing a gene regulation network by using accurate expression kinetics. Proc Natl Acad Sci U S A 99: 10555–10560. 12145321 10.1073/pnas.152046799 PMC124972

[8] Lam FH, Steger DJ, O'Shea EK (2008) Chromatin decouples promoter threshold from dynamic range. Nature 453: 246–250. 10.1038/nature06867 18418379 PMC2435410

[9] Sharon E, Kalma Y, Sharp A, Raveh-Sadka T, Levo M, Zeevi D, et al (2012) Inferring gene regulatory logic from high-throughput measurements of thousands of systematically designed promoters. Nat Biotechnol 30: 521–530. 10.1038/nbt.2205 22609971 PMC3374032

[10] Dadiani M, van Dijk D, Segal B, Field Y, Ben-Artzi G, Raveh-Sadka T, et al (2013) Two DNA-encoded strategies for increasing expression with opposing effects on promoter dynamics and transcriptional noise. Genome Res 23: 966–976. 10.1101/gr.149096.112 23403035 PMC3668364

[11] Eser U, Falleur-Fettig M, Johnson A, Skotheim JM (2011) Commitment to a cellular transition precedes genome-wide transcriptional change. Mol Cell 43: 515–527. 10.1016/j.molcel.2011.06.024 21855792 PMC3160620

[12] Zeevi D, Sharon E, Lotan-Pompan M, Lubling Y, Shipony Z, Raveh-Sadka T, et al (2011) Compensation for differences in gene copy number among yeast ribosomal proteins is encoded within their promoters. Genome Res 21: 2114–2128. 10.1101/gr.119669.110 22009988 PMC3227101

[13] Granot D, Margolskee JP, Simchen G (1989) A long region upstream of the IME1 gene regulates meiosis in yeast. Mol Gen Genet 218: 308–314. 2674657 10.1007/BF00331283

[14] Sagee S, Sherman A, Shenhar G, Robzyk K, Ben-Doy N, Simchen G, et al (1998) Multiple and distinct activation and repression sequences mediate the regulated transcription of IME1, a transcriptional activator of meiosis-specific genes in Saccharomyces cerevisiae. Mol Cell Biol 18: 1985–1995. 9528770 10.1128/mcb.18.4.1985 PMC121428

[15] Pnueli L, Edry I, Cohen M, Kassir Y (2004) Glucose and nitrogen regulate the switch from histone deacetylation to acetylation for expression of early meiosis-specific genes in budding yeast. Mol Cell Biol 24: 5197–5208. 15169885 10.1128/MCB.24.12.5197-5208.2004 PMC419861

[16] Harbison CT, Gordon DB, Lee TI, Rinaldi NJ, Macisaac KD, Danford TW, et al (2004) Transcriptional regulatory code of a eukaryotic genome. Nature 431: 99–104. 15343339 10.1038/nature02800 PMC3006441

[17] Primig M, Williams RM, Winzeler EA, Tevzadze GG, Conway AR, Hwang SY, et al (2000) The core meiotic transcriptome in budding yeasts. Nat Genet 26: 415–423. 11101837 10.1038/82539

[18] Rubin-Bejerano I, Mandel S, Robzyk K, Kassir Y (1996) Induction of meiosis in Saccharomyces cerevisiae depends on conversion of the transcriptional represssor Ume6 to a positive regulator by its regulated association with the transcriptional activator Ime1. Mol Cell Biol 16: 2518–2526. 8628320 10.1128/mcb.16.5.2518 PMC231241

[19] Williams RM, Primig M, Washburn BK, Winzeler EA, Bellis M, Sarrauste de Menthiere C, et al (2002) The Ume6 regulon coordinates metabolic and meiotic gene expression in yeast. Proc Natl Acad Sci U S A 99: 13431–13436. 12370439 10.1073/pnas.202495299 PMC129690

[20] Brar GA, Hochwagen A, Ee LS, Amon A (2009) The multiple roles of cohesin in meiotic chromosome morphogenesis and pairing. Mol Biol Cell 20: 1030–1047. 10.1091/mbc.E08-06-0637 19073884 PMC2633386

[21] Klein F, Mahr P, Galova M, Buonomo SB, Michaelis C, Nairz K, et al (1999) A central role for cohesins in sister chromatid cohesion, formation of axial elements, and recombination during yeast meiosis. Cell 98: 91–103. 10412984 10.1016/S0092-8674(00)80609-1

[22] Buonomo SB, Clyne RK, Fuchs J, Loidl J, Uhlmann F, Nasmyth K (2000) Disjunction of homologous chromosomes in meiosis I depends on proteolytic cleavage of the meiotic cohesin Rec8 by separin. Cell 103: 387–398. 11081626 10.1016/s0092-8674(00)00131-8

[23] Bishop DK, Park D, Xu L, Kleckner N (1992) DMC1: a meiosis-specific yeast homolog of E. coli recA required for recombination, synaptonemal complex formation, and cell cycle progression. Cell 69: 439–456. 1581960 10.1016/0092-8674(92)90446-j

[24] Ferrari SR, Grubb J, Bishop DK (2009) The Mei5-Sae3 complex mediates Dmc1 activity in Saccharomyces cerevisiae. J Biol Chem. 10.1074/jbc.C900023200 PMC2673244 19270307

[25] Bishop DK (2012) Rad51, the lead in mitotic recombinational DNA repair, plays a supporting role in budding yeast meiosis. Cell Cycle 11: 4105–4106. 10.4161/cc.22396 23075494 PMC3524198

[26] Kane SM, Roth R (1974) Carbohydrate metabolism during ascospore development in yeast. J Bacteriol 118: 8–14. 4595206 10.1128/jb.118.1.8-14.1974 PMC246633

[27] Sheff MA, Thorn KS (2004) Optimized cassettes for fluorescent protein tagging in Saccharomyces cerevisiae. Yeast 21: 661–670. 15197731 10.1002/yea.1130

[28] Nachman I, Regev A, Ramanathan S (2007) Dissecting timing variability in yeast meiosis. Cell 131: 544–556. 17981121 10.1016/j.cell.2007.09.044

[29] Brar GA, Yassour M, Friedman N, Regev A, Ingolia NT, Weissman JS (2012) High-resolution view of the yeast meiotic program revealed by ribosome profiling. Science 335: 552–557. 10.1126/science.1215110 22194413 PMC3414261

[30] MacIsaac KD, Lo KA, Gordon W, Motola S, Mazor T, Fraenkel E (2010) A quantitative model of transcriptional regulation reveals the influence of binding location on expression. PLoS Comput Biol 6: e1000773 10.1371/journal.pcbi.1000773 20442865 PMC2861697

[31] Tsankov A, Yanagisawa Y, Rhind N, Regev A, Rando OJ (2011) Evolutionary divergence of intrinsic and trans-regulated nucleosome positioning sequences reveals plastic rules for chromatin organization. Genome Res 21: 1851–1862. 10.1101/gr.122267.111 21914852 PMC3205570

[32] Lin Z, Li WH (2012) Evolution of 5' untranslated region length and gene expression reprogramming in yeasts. Mol Biol Evol 29: 81–89. 10.1093/molbev/msr143 21965341 PMC3245540

[33] Basehoar AD, Zanton SJ, Pugh BF (2004) Identification and distinct regulation of yeast TATA box-containing genes. Cell 116: 699–709. 15006352 10.1016/s0092-8674(04)00205-3

[34] Miura F, Kawaguchi N, Sese J, Toyoda A, Hattori M, Morishita S, et al (2006) A large-scale full-length cDNA analysis to explore the budding yeast transcriptome. Proc Natl Acad Sci U S A 103: 17846–17851. 17101987 10.1073/pnas.0605645103 PMC1693835

[35] Huh WK, Falvo JV, Gerke LC, Carroll AS, Howson RW, Weissman JS, et al (2003) Global analysis of protein localization in budding yeast. Nature 425: 686–691. 14562095 10.1038/nature02026

[36] Ghaemmaghami S, Huh WK, Bower K, Howson RW, Belle A, Dephoure N, et al (2003) Global analysis of protein expression in yeast. Nature 425: 737–741. 14562106 10.1038/nature02046

[37] Kim HD, O'Shea EK (2008) A quantitative model of transcription factor-activated gene expression. Nat Struct Mol Biol 15: 1192–1198. 10.1038/nsmb.1500 18849996 PMC2696132

[38] Leem SH, Chung CN, Sunwoo Y, Araki H (1998) Meiotic role of SWI6 in Saccharomyces cerevisiae. Nucleic Acids Res 26: 3154–3158. 9628912 10.1093/nar/26.13.3154 PMC147675

[39] Kassir Y, Adir N, Boger-Nadjar E, Raviv NG, Rubin-Bejerano I, Sagee S, et al (2003) Transcriptional regulation of meiosis in budding yeast. Int Rev Cytol 224: 111–171. 12722950 10.1016/s0074-7696(05)24004-4

